# The challenges of being imperfect: how do judges and prosecutors deal with sentencing disparity

**DOI:** 10.3389/fsoc.2024.1488786

**Published:** 2024-12-05

**Authors:** Mojca M. Plesničar

**Affiliations:** Institute of Criminology at the Faculty of Law, University of Ljubljana, Ljubljana, Slovenia

**Keywords:** sentencing, judges and lawyers, objectivity, emotions, disparity, cognitive dissonance, prosecutors

## Abstract

Legal decision-making aspires to be objective, a principle regarded as foundational to justice, public trust, and the legitimacy of legal outcomes. However, this ideal is often challenged by the reality of human judgment, which is influenced by subjective factors such as emotions, biases, and varying cognitive strategies. This paper investigates the psychological challenges faced by legal professionals in the context of sentencing, drawing on data from studies involving judges and prosecutors in Slovenia. Through workshops, interviews, and focus groups, the research highlights substantial inconsistencies in sentencing practices, even for similar offences. These disparities reveal the limits of objectivity within the judicial process, prompting legal professionals to reflect on the systemic and individual factors driving variability. The analysis focuses on how judges and prosecutors react to these discrepancies, examining a range of emotional and psychological responses—including the rationalization of decisions, the pursuit of consistency through personal “sentencing codes,” and reliance on collegial input to cope with the absence of formal guidelines. The analysis draws on concepts from cognitive dissonance theory, deliberate ignorance, emotional labour, and personality types to explore how professionals reconcile the ideal of objectivity with the imperfections of human judgment. It highlights the profound emotional toll that discrepancies in sentencing can take on decision-makers and how these emotional reactions influence their professional identity and approach to justice. By contextualising these findings within the sociology of emotions, this paper emphasises how the emotional realities of legal professionals shape their responses to perceived failures and impact their capacity to deliver justice. Ultimately, this study aims to foster a deeper understanding of the human aspects of judicial decision-making, underscoring the need for systemic reforms to mitigate disparities, provide support, and promote consistency in sentencing practices.

## 1 Introduction

Acknowledging one’s own failures is universally challenging, a truth that resonates across various personal situations and professional fields. This difficulty is particularly pronounced in professions where decisions carry significant consequences, such as law and medicine.

For legal professionals, especially judges and prosecutors, confronting and admitting imperfections in their decision-making processes is fraught with complexity. The act of sentencing involves not only applying legal principles but also navigating a labyrinth of personal judgment, societal expectations, and ethical considerations. This multifaceted process makes it essential yet particularly difficult for legal professionals to recognise and address their own shortcomings.

In the context of sentencing, the stakes are high. The outcomes of sentencing decisions profoundly affect individuals’ lives and can have far-reaching implications for justice and public trust in the legal system. The challenge of acknowledging failure in this context is compounded by the ideal of objectivity that underpins legal decision-making. While the ideal suggests that sentencing should be impartial and consistent, the reality often reveals significant disparities influenced by various subjective factors.

Thus, understanding how legal professionals deal with the recognition of their own failures and the associated emotional and professional challenges is crucial for improving both individual and systemic practices. By integrating theoretical insights with empirical data, this paper aims to provide a comprehensive understanding of how legal professionals confront and manage their own imperfections in the context of sentencing, and the implications for justice and fairness in the legal system.

This paper aims to explore the dynamics of acknowledging and responding to imperfections within the sentencing process. It begins by presenting the problem of personal fallibility in professional settings, particularly focusing on the emotional and practical challenges faced by legal professionals in sentencing. The first part of the paper provides an overview of the conceptual underpinnings of sentencing, including the ideals of objectivity and the complexities of personal and systemic factors that influence sentencing decisions. It also examines how professionals handle failure and the emotional toll associated with acknowledging imperfections. Second, the methodology is discussed, followed by a portrayal of research conducted in Slovenia, highlighting the observed disparities in sentencing and the reactions of judges and prosecutors to evidence of their own inconsistencies. This section details the emotional responses, coping strategies, and the role of deliberate ignorance in managing the recognition of imperfections. Next, the discussion ties together the theoretical and empirical findings. It explores how the personal and systemic challenges of acknowledging imperfections affect legal professionals, and discusses the implications for sentencing practices. The discussion will also address how professionals’ reluctance to embrace systemic reforms and their development of personal guidelines reflect broader themes of managing failure and seeking improvement.

## 2 Two backstories

To fully understand the challenges faced by legal professionals in the context of sentencing, it is essential to consider two interrelated narratives: the conceptual framework of sentencing itself and the broader human experience of dealing with failure. Sentencing is not merely a technical process of applying the law; it is deeply intertwined with complex social, psychological, and philosophical dimensions. The act of sentencing requires judges and prosecutors to navigate a web of legal principles, societal expectations, and personal judgments, often under the pressure of achieving an ideal of objectivity that is difficult, if not impossible, to attain. At the same time, the professionals involved in this process are human beings who must grapple with their own imperfections and the emotional toll that comes with making decisions that have profound consequences for others.

By examining both the conceptual underpinnings of sentencing and the ways in which individuals cope with failure, we can gain a more comprehensive understanding of the emotional and professional challenges that judges and prosecutors face. This dual focus allows us to see not only the structural factors that shape sentencing decisions but also the deeply personal struggles that influence those who administer justice.

### 2.1 Objectivity and sentencing

Sentencing, the process of deciding on the appropriate punishment following a criminal procedure, is undoubtedly one of the more visible phases of administering justice (Ashworth, 2015; Morgan and Clarkson, 1995) and is often said to be one of its most challenging parts (Ashworth, 2015; Maroney, 2012). Moreover, sentencing falls simultaneously at the end of the criminal proceeding and the beginning of the penal experience, thus combining very different conceptual fields. Philosophical, sociological, historical, and, importantly, psychological aspects of punishment are added to this equation, consequently producing a process that is as complex as it is important (cf. Tata, 2020).

Ideally, sentencing should be an objective process, grounded in legal principles and devoid of personal bias. However, empirical studies have consistently shown that sentencing decisions are influenced by a range of subjective factors, from personal experiences to cognitive biases (Dhami et al., 2015; Maroney, 2012).

Many authors point out that sentencing is not a solely rational (Ashworth, 2015; Lovegrove, 2006) or a solely objective process. The judge’s decision is predetermined by more or less detailed statutory or other criteria. Retorting to reason, the judge determines whether a more or less severe form of criminal offence has been committed, which mitigating or aggravating circumstances are present, what is the past case law in similar cases, and similar factors that shape the court’s final decision. However, the final decision on the sentence is much more than just a mathematical operation, a sum of rationally evaluated factors. The synthesis of all these factors requires surpassing a purely rational level and requires a certain intuitive knowledge, which, along with a rational reflection, offers a final decision. Dhami et al. (2015) refer to this concept as “quasirationality”—requiring the sentencer to work in the middle ground between the analytic and intuitive modes of cognition. It is as complex as it sounds; understanding everything it entails seems like a never-ending endeavour (Marder and Pina-Sánchez, 2020; Ulmer, 2012).

The question of objectivity in sentencing most typically comes through as the exploration of disparity. Disparity in sentencing is the occurrence of unwanted and unwarranted differences in sentencing that we can usually attribute to offenders’ personal characteristics. It is thus the opposite of sentencing consistency (Pina-Sánchez, 2015)—which still allows for different sentences to be passed. However, these differences arise from legally and morally acceptable circumstances (such as a different level of the defendant’s culpability and a different severity of crime). Inquiries into disparity in sentencing range from complex studies into its prevalence to more psychologically oriented studies into why it occurs (Sporer and Goodman-Delahunty, 2009). On the one hand, we aim to measure the extent of disparity—between judges and between courts or regions within a given jurisdiction. Generally, most such studies’ prevailing result is that there is much more disparity than expected and that even measures taken to limit it (such as restricting sentencing discretion) rarely lead to the desired outcome (Drápal, 2020; Scott, 2010). On the other hand, disparity is dissected and tied with potential prejudice and bias towards gender, race, nationality, or other personal characteristics (Cho and Tasca, 2018; Freiburger, 2010; Mustard, 2001; Skeem and Lowenkamp, 2016; Van Wingerden et al., 2014)—which would be the very opposite of objective legal decision-making.

Significant efforts have been made in the past against increasing evidence on sentencing disparity to curtail its detrimental effects or eradicate it completely. In this context, the main area of interest is structuring sentencers’ discretion, thus answering the question of how much room is/should there be for the decision-maker to form the sentence (Brown, 2019; Drápal and Plesničar, 2023; Roberts, 2009). Legal systems vary from offering sentencers limited discretion (e.g., narrow sentencing tables, mandatory sentences) through offering ways of structuring discretion (e.g., sentencing guidelines, judicial self-regulation, statutory ranges) to broad discretion (e.g., vague statutory ranges). The extremes are less likely to be found in modern systems, but there are many different and constantly changing variants (Kurlychek and Kramer, 2019). Moreover, discretion in sentencing does not lay only in the hands of the judge but is distributed—sometimes haphazardly—to other participants as well, most notably the prosecution (Bushway and Forst, 2013).

Furthermore, given the complexity of the decision and the intersection of disciplines at which sentencing lies, sentencers might be even more prone to typical decision-making mistakes than in other decisions throughout the legal process. Most notably, the effects of cognitive bias, especially anchoring on sentencing, have been explored and confirmed. Judges seem to be as prone to cognitive bias as ordinary people, and anchoring—basing your decision on a previously randomly set anchor—substantially impacts their decisions (Guthrie et al., 2007; Mussweiler et al., 2012; Rachlinski and Wistrich, 2017). Moreover, the context of emotions in the courtroom has been the basis of some pivotal studies, changing our understanding of how (non-)emotional judges and other professionals are or should be (Anleu et al., 2016; Jamieson and Tata, 2017; Karstedt et al., 2011; Maroney, 2012).

Finally, it is important to recognize that one of the central goals of sentencing is to individualise the punishment to fit both the defendant and the specifics of the crime (Bierschbach and Bibas, 2016; Frase, 2001; Plesničar, 2013). This principle of individualisation is rooted in the idea that justice requires more than a one-size-fits-all approach; it demands careful consideration of the unique circumstances surrounding each case. Factors such as the defendant’s background, the severity of the offence, and the presence of mitigating or aggravating circumstances all play crucial roles in shaping a sentence that is both fair and appropriate. Individualisation is emphasised differently in different systems, with continental European systems being quicker and more open about it being a guiding principle in sentencing than common law systems (Drápal and Plesničar, 2023).

However, a delicate balance exists between ensuring that sentencing is sufficiently individualised and avoiding the pitfalls of arbitrariness—opening doors to disparity. On the one hand, if sentencing guidelines and statutory frameworks are too restrictive, they can stifle the judge’s ability to tailor the sentence to the nuances of each case, leading to outcomes that may seem unjust or overly rigid. On the other hand, if these guidelines are too lenient or vague, they can open the door to excessive discretion, which may result in inconsistent and potentially biased sentencing decisions. Thus, the challenge lies in finding the right balance—a framework that provides clear and consistent guidance while still allowing judges the flexibility needed to account for the individual characteristics of the defendant and the crime. None of the existing sentencing systems find this balance perfectly, but some do it better than others. Slovenia, which we will use as an example in the discussion below, falls within the second group.

### 2.2 Dealing with imperfection in a legal and sentencing setting

Professionals across various fields are often held to high standards, with little room for error. In professions where decisions have significant consequences, such as law or medicine, the emotional toll of recognising one’s imperfections can be particularly heavy (Gawande, 2003; Nice, 2001). This is especially true in the legal profession, where the expectation of delivering just and fair decisions is paramount.

Just and fair is, however, very hard to define. Our modern legal systems often settle for the decision that results from an impartial and/or objective decision-making process. The difference between the two notions is not well defined (Breda, 2017; Dyrda and Pogorzelski, 2011) and not strictly necessary for the sake of our discussion. Impartiality is often viewed as an ethical and legal principle, where personal beliefs, past experiences, and personal connections should not influence the decision-making process. It is about maintaining neutrality and avoiding bias in judgments (Lucy, 2005; Papayannis, 2016). On the other hand, objectivity is broader and perhaps harder to define. Looking for a common thread among the many attempts to define it, we can see objective decision-making as a process of reaching conclusions or choices that are devoid of personal bias, emotions, and subjective opinions (Grossi, 2019). Such decision-making implies basing the final decision on the impartial evaluation of factual information and relevant data while using established rules and well-defined criteria. Like impartiality, objective decision-making would seek to minimise the influence of personal preferences, prejudices, and extraneous factors that could sway the outcome in a particular direction (Breda, 2017; Brink, 2000; Grossi, 2019; Leiter, 2000). Additionally, some definitions pose that objectivity involves emotion management and empathy to ensure that decisions are based on facts and evidence rather than personal feelings (Blix and Wettergren, 2019).

We have long known that objectivity is an ideal that is hard to achieve. Decades of empirical research have put it under significant scrutiny, resulting in findings that refute the idea of (total) objectivity in legal decision-making (Kapardis, 2009; Klein and Mitchell, 2010). Many of these studies show that legal decision-making is just as prone to cognitive bias, prejudice and errors as other contexts of decision-making. Studies have thus shown strong evidence of judges succumbing to anchoring effects, hindsight bias, confirmation bias, framing effects etc. (Guthrie et al., 2007; Kahneman et al., 2021; Meterko and Cooper, 2021; Mussweiler et al., 2012; Rachlinski and Wistrich, 2017). It seems, as Schauer (2010, p. 103) puts it, that it is “the judge as a human being and not the judge as judge or the judge as lawyer—that has the greatest explanatory power in accounting for judicial behavior.”

Such insights occasionally come even from judges themselves, who admit through various writings their own or their colleagues’ approaches to judging that deviate from the expected idea of objectivity. Such admissions often come from prominent judges in systems where the judicial function holds more autonomy and even character, e. g. the US Supreme Court (Wrightsman, 1999), but we can find them elsewhere too. Continental judges are less likely to circulate such ideas widely, but it may occur: a Slovenian former Supreme Court judge, for example, explains:

*“This was a given among the judges, we were aware that there is no such thing as a completely impartial judge and trial, one where, with our eyes closed, following only the flow of argumentation, we will finally find the (objectively already existing?) correct solution. More so, because no such correct solution exists that could be found through simple intellectual research. It is true that judges do not find the right solution, they create it. This is all the more true if the decision is to be not only ‘right’ but also just”* (Testen, 2019, pp. 11–12).

Regardless of such testimonies and, more importantly, rigorous research, modern legal systems continue to operate under the assumption that objectivity is the norm in legal decision-making. The way law is designed in legislation, taught to law students, and presented to professionals and the public all imply that there is no room for subjective elements that could taint the objective façade of the legal norm. This is perhaps even more true in continental legal systems. These have evolved since Montesqueiu’s position on judges being solely “la bouche qui prononce les paroles de la loi,” but still act on this premise as a starting point.

This feigned ignorance of what lies underneath, however, has its purposes. In other contexts, “deliberate ignorance” has become an interesting notion, giving academic weight to Thomas Grey’s insightful point that ignorance is bliss. Hertwig & Engel (2021, p. 360) have defined it as “the conscious individual or collective choice not to seek or use information or knowledge.” They delineate a plethora of reasons why this may occur, listing, for example, deliberate ignorance as an emotion-regulation and regret-avoidance device, a suspension- and surprise-maximization device, a performance-enhancing device and, most relevantly, an impartiality and fairness device (Hertwig and Engel, 2021).

This concept seems relevant to our sentencing context, where judges and prosecutors may consciously or unconsciously engage in deliberate ignorance as a coping mechanism to manage the emotional burden of their decisions. By selectively ignoring certain imperfections or uncertainties, made known, for example, by numerous studies showing sentencing disparities, they can maintain a sense of impartiality and fairness, which is essential for upholding the legitimacy of their role.

However, this strategy might also allow them to avoid the paralysing effects of doubt and regret that might arise from acknowledging the full complexity and subjectivity of their decisions. In this way, deliberate ignorance may serve as both a protective device and a functional necessity, enabling legal professionals to carry out their duties while preserving their professional identity and emotional well-being (Eigen and Listokin, 2012).

However, when confronted with the realisation of their own imperfections, legal professionals often experience a profound emotional response. Research in the sociology of emotions has shown that professionals in general often experience guilt, shame, and anxiety when they perceive themselves as falling short of these expectations (Scheff, 1994). In the context of legal decision-making, these emotions are compounded by the knowledge that their decisions can have life-altering consequences for the individuals involved (Hagan and Kay, 2007; Krause and Chong, 2019). These emotions are not merely abstract; they manifest in very real ways as professionals grapple with the implications of their actions.

Immediate proof of imperfection, such as empirical evidence of bias or error in decision-making, can be particularly unsettling. Their reactions to such evidence may reflect distinct personality types as developed by Dattner and Hogan (2011), which can significantly impact their response to failure and blame. Extrapunitive responses may involve defensive behaviours where legal professionals shift blame to external factors or others involved in the case, downplaying the significance of the evidence and avoiding personal accountability. Impunitive responses may manifest as denial of the problem’s existence or their role in it. This could involve dismissing or rationalising away evidence of errors or biases, thereby avoiding confrontation with the reality of their imperfections. Intrapunitive responses may lead to excessive self-blame and self-criticism. Judges and prosecutors with this tendency might experience intense self-doubt and anxiety, losing confidence in their abilities even when the evidence suggests that their mistakes are minor or part of the inherent complexities of legal decision-making.

Some professionals may even experience a form of cognitive dissonance, where they attempt to reconcile their self-image as fair and objective with the reality that their decisions are influenced by subjective factors (Festinger, 1957). Festinger’s original idea that individuals feel psychological discomfort when they hold two competing cognitions (e.g., perceptions, attitudes, behaviours, feelings) was further developed and tested by various authors (Cooper, 2019). In the context of judges and prosecutors, the action-based model of cognitive dissonance offers a particularly useful lens through which to understand how these professionals deal with the conflicts between their ideal of impartiality and the subjective nature of sentencing. This model extends the traditional understanding of cognitive dissonance by emphasising that dissonance not only causes discomfort but also interferes with effective action (Harmon-Jones and Harmon-Jones, 2018; Harmon-Jones and Harmon-Jones, 2023). For judges, the experience of dissonance can stem from the recognition that their sentencing decisions—ideally based on objective criteria—are influenced by various subjective and situational factors. According to the action-based model, such conflicting cognitions can disrupt their goal of delivering just and consistent sentences, thereby hindering their ability to act effectively in their role as impartial decision-makers. To alleviate this discomfort and regain effective action, judges may be motivated to re-evaluate their approach to sentencing, seeking ways to align their behaviours more closely with their self-image as fair and objective. In this sense, the drive to resolve dissonance may lead some judges to engage in self-reflection and strive for improvements in their decision-making processes (McGrath, 2017). They might, for instance, reconsider their reliance on heuristics or subjective factors, or they may turn towards collegial input or newly proposed guidelines to enhance the consistency of their decisions. In the most optimistic view, by aligning their cognitions with their professional goals, they may ultimately improve their ability to deliver sentences that are fair, consistent, and in line with the principles of justice.

Ultimately, the way legal professionals deal with imperfection and its immediate proof varies widely, but it often involves complex emotional and cognitive processes. Some may adopt strategies of deliberate ignorance, as previously discussed, to shield themselves from the full emotional impact of their decisions. Others may seek to improve their decision-making processes through self-reflection and additional training, allowing them to foster a deeper understanding of their professional conduct and the emotional impacts of their work (Gibbons, 2019; Maroney and Gross, 2014; Sheppick, 2024; Spencer and Brooks, 2019). At the same time, they may try to process their imperfection through peer support and feedback, though I was unable to find supporting research on this issue (cf. Roach Anleu and Mack, 2014; Završnik et al., 2023). However, paralleling findings in the medical field, discussing incidents and mistakes in a supportive environment is paramount to better one’s performance. Such environments allow professionals to disclose their mistakes, discuss them with colleagues, and accept their fallibility, which is vital for their mental well-being and professional integrity (O’Beirne et al., 2012). Finally, some professionals may be unable to react to the issue in a meaningful way, leaving them despondent, professionally and personally frustrated and stressed (Edwards and Miller, 2019; Resnick et al., 2011; Schrever et al., 2021). Regardless of the approach, it is clear that the acknowledgment of imperfection adds significantly to the psychological and emotional toll that working in criminal justice necessarily entails. It unavoidably entails tiresome emotional work (Hochschild, 1983) and is a significant and challenging aspect of the professional lives of judges and prosecutors.

## 3 Methodology

### 3.1 Methodological background

Despite growing research on sentencing disparities globally, many (even European) countries remain underexplored. Slovenia, a youngish European democracy, has only recently begun examining these issues, with limited studies available (Drápal and Plesničar, 2023). The Slovenian sentencing system, characterized by broad statutory ranges and a focus on individualisation, compares with the more restrained penal models of Scandinavian countries (Dünkel, 2013; Flander and Meško, 2016; Plesničar and Drobnjak, 2019). This system emphasises tailoring sentences to the offender and the offence, allowing for extensive judicial discretion (Plesničar, 2013). The main sentencer in the system continues to be the judge—typically a single professional judge or, in rare, more serious cases, a mixed panel of professional and lay judges. However, the role of prosecutors has become more pronounced in recent decades—first by requiring them to propose adequate sentences in all cases and later by introducing plea bargaining where the prosecutorial decision on the sentence is merely confirmed by the judge (Plesničar et al., 2023a).

On one hand, the system has led to surprising parsimony in sentencing practices; however, recent studies suggest that this leniency may come at the cost of increased sentencing disparity. In an experiment using vignettes, we explored how biases related to gender, socioeconomic status, ethnicity, and appearance influenced sentencing decisions. Contrary to international findings, we did not find clear evidence of these biases but observed substantial variability in sentencing outcomes, consistent with Kahneman et al.'s (2021) concept of “noise”—unwanted inconsistencies in decision-making. This variability was evident in cases where judges and prosecutors imposed vastly different sentences for the same offence (see more details in the next section).

Our broader study, analysing 1,473 cases across 11 offence types, confirmed that such inconsistencies are also present in actual sentencing. We found significant variability in both the length of prison sentences and the imprisonment threshold, with a high percentage of unexplained variability, suggesting substantial disparity (Plesničar et al., 2023a).

A third study focusing on sexual offences employed a qualitative approach to further investigate sentencing variance. The findings indicated marked differences in how courts evaluated similar factors, with some courts treating mitigating circumstances inconsistently. This qualitative analysis corroborated the quantitative results, highlighting unequal treatment and inconsistent application of sentencing principles (Plesničar et al., 2023b).

This paper is built on the background of these studies. We wanted to investigate how facing these results—proof of failing at being objective at both a very individual, personal level and at the systemic level—impacted the professionals and how they reacted to it.

### 3.2 Data collection and analysis

The data used in this paper were gathered through three primary methods. The participants were selected based on their roles within the legal system and their experience with sentencing, ensuring representation from both judges and prosecutors involved at different levels of the judicial process.

First, we conducted observations during workshops involving separate groups of judges and prosecutors. These workshops were designed to explore sentencing disparities and other forms of bias. Participants were tasked with selecting sentences for various cases, first individually and then as part of random panels of three professionals, and their decisions were anonymously processed and shared with the group. This setup allowed us to observe how participants engaged with and responded to evidence of significant variations in their personal sentencing choices.

The three judicial workshops were organised within the Judicial Training Centre as part of regular professional development education offered to judges in 2018. Participants had to apply to participate, but all judges had access, and three iterations were conducted specifically for criminal judges. Both first-instance and appellate judges participated, along with a smaller group of judicial assistants who perform judicial tasks within the system. The judicial workshops included a total of 96 participants: 83 women and 13 men (reflecting the general gender structure in the Slovenian court system), consisting of 81 judges and 15 judicial assistants, with an average of 14.2 years of experience.

The three prosecutorial workshops were organised in cooperation with the Supreme State Prosecutor’s Office and were held via Zoom in 2021. They were offered to all prosecutors, covering the entire geographical scope of the system. Both junior and senior prosecutors participated, resulting in a total of 66 participants: 46 women, 17 men, and 3 without gender data, with an average of 9.1 years of experience. This ensured a comprehensive representation of perspectives within the prosecutorial service.

This approach ensured diversity across different regions and court levels, providing a comprehensive perspective on sentencing practices. However, the voluntary engagement of participants might indicate a stronger-than-average interest in these topics, which could be a potential limitation for these results. It suggests that those who participated may be more interested in and reflective about these issues, possibly limiting the generalisability of the findings. Nonetheless, given the difficulty of accessing legal professionals for experimental research, this was considered the best feasible approach.

During these workshops, five vignettes depicting fictional yet realistic cases were used to evaluate sentencing decisions, and the vignettes were developed based on typical cases seen in Slovenian courts. They were tested through limited cognitive interviews with professional judges and other legal professionals and validated by a panel of senior legal professionals at the Institute of Criminology to ensure their realism and relevance to actual practice. The professionals at each workshop were divided into two random groups and were randomly assigned the five vignettes, each containing one key variable (gender, social status, ethnicity, appearance, and prosecutorial suggestion). This setup allowed us to examine how each variable influenced sentencing decisions and to understand the degree of variability across participants.

Presenting the results in more detail would exceed the scope of this article, particularly given the complexity of the interactions among variables and the volume of data gathered. However, key results indicate significant disparity, with large standard deviations observed across different groups. For example, substantial variability was found within the sentencing decisions made by judges and prosecutors, with no statistically significant differences in responses to variables such as social status, gender, and ethnicity. Specifically, standard deviations for sentences ranged from 20 to 50% within the groups tested, pointing to a high level of inconsistency in sentencing outcomes.

During the workshops, one of the researchers took observational notes, which were later checked for consistency and copy-edited.

Secondly, six follow-up semi-structured interviews were carried out in 2020 with three judges who participated in the workshops and three who did not attend. The interviews were designed to be broader in scope, exploring participants’ attitudes towards punishment in general, while also capturing in-depth reflections on operating in an imperfect sentencing system. The interview questions were developed to probe the impact of personal and systemic factors on sentencing and included questions such as: “How strict do you consider yourself when sentencing? More or less strict than the average judge? Do you feel that sentencing policy is consistent? How could it be made more consistent?” and “Is the approach to this question different at the start of a judge’s career compared to after some time? How long does this period of ‘adjustment’ last?” This semi-structured approach allowed flexibility, enabling interviewees to elaborate on issues they found significant, while ensuring that core themes related to disparity and objectivity were covered. The interviews were recorded and transcribed.

Thirdly, we conducted focus groups with two groups of prosecutors and a separate group of senior judges and prosecutors via zoom, to discuss strategies for addressing the disparities identified in our study. The focus groups were organised in collaboration with the Supreme State Prosecutor’s office. All prosecutors received an invitation to participate and were selected on a time-of-application basis. These discussions aimed to gather insights on potential solutions and improvements for managing sentencing inconsistencies. Focus groups were chosen to foster a collective discussion dynamic, where participants could interact and build on each other’s ideas. The prosecutors were purposefully separated by seniority to ensure they felt comfortable discussing with peers of similar experience. The specific questions posed during these discussions included: “How do you decide on what sentence you will recommend?” and “What kind of support would you need to make better recommendations?” The focus groups were not recorded due to a reluctance on the side of the participants. Instead, two sets of notes were taken to record the discussion in as much detail as possible. The notes were later compared for consistency and copy-edited.

Participants were assured confidentiality, and all identifying information was anonymised to protect their privacy. Informed consent was obtained from all participants, and the research adhered to ethical guidelines regarding the management of sensitive data.

The interviews and focus group discussions were analysed thematically, alongside observational notes from the workshops. Thematic analysis (Braun and Clarke, 2019) involved systematically coding the data to identify key themes related to professionals’ reactions to evidence of disparity, their explanations for its occurrence, and their personal and systemic coping strategies and solutions. This approach allowed for a deeper understanding of how legal professionals perceive and manage imperfections in their sentencing decisions. NVivo software was used to assist in organising and retrieving coded data, ensuring a systematic approach to identifying patterns and themes.

The decision to use a qualitative approach was driven by the need to explore the subjective experiences of legal professionals more in-depth, offering insights into their emotional and cognitive responses to the challenges of their roles. The combination of observational, interview, and focus group data provided a comprehensive perspective, capturing both individual reflections and group dynamics related to the emotional and professional challenges of sentencing.

## 4 Observing reactions to acknowledging imperfections

### 4.1 The reckoning

Ideally, professionals should be aware of the outcomes produced by the criminal justice system. However, their understanding may be constrained by the transparency of the sentencing system and the scope of available data. In a system like Slovenia’s, where there is a lack of aggregated or disaggregated data on average sentences or sentencing practices, professionals’ knowledge may be confined to their own limited experiences and case-specific observations. This limitation can affect their ability to fully grasp broader patterns and trends in sentencing.

On the other hand, knowledge of disparities in sentencing is widespread and in a system as small as the Slovenian one, professionals often feel that their personal experience confirms it. Prosecutors, especially, have expressed a firm belief that sentencing practices vary significantly between different courts, with a common perception that courts in the Northeast are notably more punitive compared to those in the Southwest (which was, to a small extent, confirmed by the studies) *(Focus Group 1,3)*.

However, this perception can also be found among judges,

*Judge 5: "Yes, yes, it seems to me that sentencing is even stricter here in [bigger court] compared to [smaller court]."*

*Judge 2: " Unfortunately, determining the severity of sentences [in our system] is very subjective."*

*Judge 1: "Yes, this range is noticeable among us. Certainly, it is. It is noticeable, for example, how informally things are handled. Initially, it is mostly the prosecutors or lawyers who know this best. Some [judges] are very strict, some are more lenient, some are more inclined towards acquittals. They are just stricter with charges, you know."* ^1^

who sometimes pointed back at the prosecution itself:

*Judge 1: "Yes, their [prosecutorial] sentencing policy is inconsistent."*

*Judge 4: "Sentencing in [my court], based on criminal records of offenders who [have committed crimes] in other areas as well, is, at least according to the records, stricter than sentencing in [other town]. Or, I don’t know, [other town]… or [other town]. For very similar offences, for example, where one sentence might be one month in prison, another might be two months or three months, the prosecutor here wanted six months as a starting point.”*

In none of the settings where we discussed it at a general level, this knowledge of inconsistent sentencing was something new or surprising. Some of our collocutors expressed disappointment and were more bothered by it than others *(Focus Group 3)*, but generally at least partly attributed it to the notion of individualisation itself.

*Judge 5: "Well, some differences are inevitable, I think; these are not things that can be completely standardised. It’s also utopian to expect that, you know! However, there shouldn’t be significant differences, you know."*

*Judge 4: "No, no. I don’t think it interferes with autonomy. I am firmly of the view that… legal interpretation must be socially specific, that is, tailored to a particular environment, and that differences in sentencing policies across different environments are not inherently problematic."*

However, this spot of bother grew when the disparity was either made more explicit or brought closer to the respondents. The experimental study was conducted as a workshop and served both research and educational purposes. We presented judges and prosecutors with immediate feedback on their sentencing choices for hypothetical cases, where the extent of variation among their decisions was obvious an obviously staggering. For example, in the most divergent case, where they needed to sentence an offender for the crime of aggravated bodily harm resulting in death, the sentences they chose ranged from a 6-month conditional sentence to 6 years in prison.

*Judge 5: "We were just at your seminar […] if you remember. […] We worked in groups [and] one colleague, [in the case of the] female offender, proposed a significantly higher sentence than I did […]. Generally, he proposed harsher punishments than I did in all the cases. I was on the complete opposite end […]."*

The personal involvement in these decisions led to expressions of shock, disbelief, disappointment and even shame—emotions not observed with regard to the more general knowledge of sentencing disparities. When we observed their interactions in panel settings, some of them had animated discussions, at times even with raised voices and strong hand gesturing. When presented with the results showing large disparities, they shook their heads, murmured and whispered to each other with surprised facial expressions; some sighted loudly, and one participant loudly exclaimed: “This cannot be true!” *(Observation notes)*. The direct engagement in sentencing seemed to reinforce their own views on the appropriate sentences—which they had reinforced in the panel setting of the vignette—while simultaneously amplifying their discomfort upon discovering that their colleagues’ views differed significantly.

When presented with the findings from our other studies, prosecutors expressed concern and disappointment, but were much less personally affected by the results *(Focus groups 1–3)*. Furthermore, not everyone felt discomfort even in the experimental setting, one judge, for example, expressed that she knows her sentences differ significantly from those of her colleagues:

*Interviewer: "And … are you okay with this? I mean, do you feel comfortable with it? Have you ever found it problematic?"*

*Judge 5: "No, because that’s how I see things, that’s how I interpret them. And I believe I explain them well, that I am thorough in this regard. Sometimes I succeed, but not often, not always, I would say. But it does happen, you know, that in the majority of cases, my decisions in this area are changed [by higher courts], usually resulting in increased sentences. It has happened, of course, in 23 years, that sometimes my sentences were reduced, but I don’t think there were even ten such cases in 23 years."*

### 4.2 The reasoning

In exploring the reasons behind the observed disparities in sentencing and seeking potential solutions, many judges and prosecutors expressed a sense of abandonment in their decision-making processes. They highlighted a lack of systemic support and guidance, which significantly impacts how they approach sentencing.

Several professionals noted that, apart from occasional collegial advice, there is no structured guidance or data to inform their sentencing decisions. The absence of comprehensive, aggregated sentencing data and the lack of a formal learning environment contribute to this sense of abandonment. Judges and prosecutors often feel that they operate without adequate systemic tools to counteract disparities, acknowledging that generalised norms are insufficient to address the nuances and complexities of individual cases.

*Judge 3: "It’s challenging because, in practice, it is very difficult to determine a sentence for specific cases without some reference points. I usually… and this is… as a judge, it was one of the hardest things I faced at the beginning. I remember the first few times I had to impose sentences; I really had a lump in my throat. I clearly remember the first case where I sentenced someone to, I think, two years and six months. I was preparing and justifying that sentence. It was for a gentleman who had drug problems and so on. I had a lump in my throat […] It is frustrating because you don’t have a reference tool or a measuring instrument to guide you in these initial, in your initial cases. For someone who has prior convictions, you can look at their record—say, for serial offenders or habitual criminals. For thefts and such, you check their criminal record and see what they’ve received before. You think, ‘Okay, since they’ve done this five times before, I can’t give them less than they received previously.’ So you might go higher and then it builds up from there."*

This quote illustrates the profound emotional impact of sentencing, particularly in the absence of clear guidelines or structured reference points. Judge 3’s use of the phrase ‘a lump in my throat’ captures the anxiety and personal distress they felt when making the first few sentencing decisions, highlighting the psychological toll of imposing life-altering consequences on others. The reference to a ‘measuring instrument’ further underscores the judge’s struggle with the lack of clear and standardised tools to guide their decisions, adding to the emotional burden. This emotional turmoil can be linked to broader theoretical frameworks on guilt, shame, and anxiety in professional settings, particularly in high-stakes decision-making contexts (Scheff, 1994; Hagan and Kay, 2007; Krause and Chong, 2019). For judges, this dual pressure of having to balance fairness and consistency, while also managing their own emotional responses, creates a situation where they may feel ‘left on their own’ in the decision-making process. This isolation, combined with the weight of responsibility, often fosters a fear of making a wrong decision, as illustrated by Judge 3’s statement. This fear is not only about the potential for personal mistakes but also about the broader implications of those mistakes, including the possibility of public scrutiny, shame, and professional consequences.

Similarly, one of the prosecutors expressed deep frustration and incredulity over the fact that he cannot access prosecutorial files in similar cases within their database due to data protection restrictions. He felt that this lack of access severely undermines his ability to perform effectively, as it hinders his capacity to propose appropriate and consistent sentences *(Focus Group 3)*.

This observation from the prosecutor adds another layer to the emotional challenges faced by legal professionals, specifically in the context of inconsistency and frustration with the system. Like Judge 3, who expresses the emotional toll of making sentencing decisions without clear guidelines, the prosecutor’s frustration stems from being unable to access critical information that could help ensure consistency and fairness in their decisions. This lack of access to prosecutorial files underscores a key point from the judge’s perspective: without the appropriate tools or structured support, both judges and prosecutors are left to rely on their own judgment, which can lead to inconsistency and a sense of isolation in the decision-making process.

In both cases, there is a struggle with the absence of standardised reference points, which can leave professionals feeling unsupported and vulnerable to making errors. For judges, the fear of making a wrong decision carries emotional weight due to the life-altering consequences of sentencing. Similarly, the prosecutor’s frustration reflects the emotional toll of not being able to perform their role effectively, compounding their feelings of inadequacy. Both professionals are left to navigate the complexities of sentencing, but without the institutional resources or frameworks that could provide clearer guidance.

However, others seem quite unbothered with the lack of such institutional support and are happy to adhere to the personal codes that they themselves develop, as long as they are able to justify them to themselves:

*Judge 1: "I really don’t have any issues with this. You know, when I look back, my sentences are always approximately the same. It’s hard to say that there are deviations or anything like that. For instance, a robbery is generally around four years. That’s the starting point, you see. Because these are serious offences. Then, well, I don’t know. If you ask me, I can generally recall that all similar cases are treated approximately the same, and I calibrate them similarly, even though I can't describe it to myself. People often ask me how I make my decisions. I don’t know. It’s based on each individual case, within a range, but also how I apply it to each person."*

*Judge 4: "I don’t know. [Laughs] I look at those with longer criminal records, review their case files, and see what sentences they received… then I go by some sort of intuition. I’m not sure if it bothers me that I don’t have completely established criteria or not. But when I consider how to handle this issue, I think that… one starting point could be that I have no pre-set criteria at all, and another could be having completely rigid criteria. One of the prosecutors I never agree with is at the extreme end; they have rigid criteria that they don’t deviate from, regardless of the specifics of the individual. Even if… I’m not just talking about the impression someone makes when they come into the courtroom, but also the circumstances in the case file that could affect the decision. But for them, nothing influences it. It’s always the same: if I think it should be conditional, it’s conditional, and that’s what I propose in the order; otherwise, it’s prison. And then, they have tables for how much prison time for each case. On the other hand, you only have the legal framework, and I don’t think that’s right. I believe I am somewhere in between these two extremes, in the sense that I consider… at this moment, I think about what I usually impose for such individuals or for these types of offences and what seems appropriate based on the impression someone has made on me, and the specifics of the offence. Or I mix in what might be suitable for the particular case.”*

These quotes reveal different approaches to sentencing and demonstrate the varying levels of comfort legal professionals have with the lack of formal guidelines or institutional support. Judge 1, for example, expresses confidence in the consistency of their sentencing, noting that while they cannot fully articulate their reasoning, they rely on a personal, calibrated approach. This ‘starting point’ method appears to offer them some security, as it allows for consistency across their cases, even though it might not strictly adhere to clear-cut criteria. The reliance on a personal ‘range’ for each type of offence, alongside the individualisation of each case, shows an acceptance of subjectivity. However, judge does not question it in a broader context, considering fairness across cases and the transparency of their reasoning.

Judge 4 offers a more reflective perspective on their approach, acknowledging the absence of rigid criteria but also comparing it with more structured approaches. Their method is seen as more flexible and tailored, considering the nuances of each case. The reference to ‘intuition’ as part of their decision-making process reveals the informal nature of their approach, allowing for subjective discretion. However, this approach also carries potential risks, as it might lead to inconsistencies and disparities, as seen when they contrast their own methods with a prosecutor’s rigid application of sentencing tables. Here, Judge 4 reveals an awareness of the shortcomings of both extremes: a strict, table-based approach versus the more flexible, individual-driven one. This highlights the tension between flexibility and consistency in sentencing. While Judge 4 seems to believe in the importance of adjusting for the particulars of each case, there is no shared framework to ensure that this subjective flexibility is consistently applied across different judges and courts.

Both quotes emphasise the internal struggle faced by judges in balancing personal judgment and fairness. While Judge 1 seems comfortable with their approach, relying on a sense of consistency based on experience, Judge 4 is more critical of the extremes and seeks a middle ground. However, both judges ultimately fall into the category of relying on personal frameworks, which can mitigate the emotional burden of sentencing but also limit accountability and transparency. In comparison to prosecutors, who are more likely to follow established guidelines (even if rigid), these judges’ methods reflect an ongoing tension between autonomy and the need for systemic structures that can foster greater consistency and fairness in the sentencing process.

In addition to Judge 3’s initial observation in this section, several other professionals also discussed the contrasts between sentencing decisions made early in their careers compared to those made later, after gaining more experience as judges.

*Judge 2: "Yes, I think that at the beginning I might have even been—now, at least in my experience, I was perhaps a bit too lenient at first. Then, after a few [appellate court decisions], I gained some insight into how the appellate court thinks and I adjusted my sentencing framework accordingly. […]*

*Interviewer: "So, you learn what is expected of you?"*

*"Yes, but I still miss, or rather, I really wish we had the German system, where for every crime there is a defined way to determine the sentence. Not as a … formula, but as parameters; this, this, this, this, and this, so you have a pretty good overview of sentencing for all offences."*

*Judge 4: “Initially, I … I started by reviewing all the files assigned to me. I looked at the criminal records and saw what kinds of sentences were handed down in [my court], and I didn’t think it was appropriate to deviate too much from expectations in that specific social context. That was a starting point for me, and then … in specific cases, I go by some kind of feeling."*

These quotes reflect the evolution of their sentencing approaches as they gain experience. Judge 2 acknowledges a more lenient approach in the early years, influenced by the appellate court’s feedback. This evolution demonstrates how external guidance, such as appellate decisions, helps shape and refine sentencing frameworks over time. The judge’s desire for more structured parameters, similar to the German system, suggests a preference for clearer guidelines to reduce uncertainty and improve consistency in sentencing. In contrast, Judge 4’s experience highlights a different aspect of the learning process. Initially, they relied heavily on the criminal records and previous sentencing practices in the court, feeling compelled to align with established norms. Over time, this approach gave way to a more intuitive decision-making process, where personal judgment played a larger role. This shift from a reliance on reference points to intuition underscores the personalisation of sentencing.

Additionally, discussing the structural features of sentencing, some frustration was expressed with the procedural structure of the legal system. Some professionals expressed dissatisfaction with how the system is designed, noting that the lack of bifurcation in trials—where the verdict and sentencing stages would be addressed separately—further complicates efforts to achieve consistent and fair sentencing. They feel that this procedural rigidity, combined with the absence of robust support systems, exacerbates the challenge of maintaining uniformity and fairness in sentencing.

This dual responsibility creates additional cognitive load and can compromise the ability to focus solely on crafting a fair and consistent sentence. Moreover, without clear separation, professionals often struggle to apply consistent standards, leading to potential disparities.

Finally, prosecutors and judges felt that some of the inconsistences in sentencing were caused by unclear legislative changes and the slow rate at which case law adapts and refines them *(Focus Group 3)*.

Overall, the search for reasons behind sentencing disparities reveals a dual perspective. Some respondents voiced profound concerns about the current system, expressing that it lacks adequate systemic support and that procedural structures fail to effectively address or mitigate the factors contributing to sentencing variability. Conversely, others acknowledged the problematic nature of sentencing disparity but argued that it cannot be quantified to a degree that would make the process straightforward or fully objective.

Overall, the search for reasons behind sentencing disparities reveals a dual perspective. Some respondents voiced profound concerns about the current system, expressing that it lacks adequate systemic support and that procedural structures fail to effectively address or mitigate the factors contributing to sentencing variability. These professionals pointed out that without clear guidance or consistent frameworks, they are left to navigate the complexities of sentencing on their own, often making decisions in isolation. This lack of institutional support not only contributes to inconsistencies but also places a significant emotional burden on judges. The weight of making life-altering decisions without clear reference points can lead to feelings of inadequacy, anxiety, and fear of making mistakes. The emotional toll is exacerbated by the high stakes involved, as judges are acutely aware that their decisions can affect individuals’ lives in profound ways. This emotional distress is compounded by the pressure to balance fairness, consistency, and individualisation, with many judges expressing that they often feel overwhelmed by the responsibility.

Conversely, other respondents acknowledged the problematic nature of sentencing disparity but argued that it cannot be quantified to a degree that would make the process straightforward or fully objective. These professionals contended that the subjective nature of sentencing, with its reliance on personal judgment, is an inherent feature of the system and that trying to impose complete consistency would undermine the flexibility needed for individualised justice. While they recognised the disparities that can arise, they also believed that such variability is an inevitable consequence of the complexity of each case. For these professionals, the emotional burden of sentencing is less about the lack of systemic support and more about navigating the tension between ensuring justice in each unique case and maintaining consistency across decisions. This perspective suggests that while the system may benefit from improvements, achieving a fully objective and consistent approach to sentencing may not be feasible or desirable without sacrificing the essential human element of judicial discretion.

### 4.3 The resolution: from the personal to the systemic

The challenges associated with sentencing disparity have led many judges and prosecutors to develop their own approaches to sentencing. In response to the lack of systemic guidance and the perceived inadequacies of the current system, professionals often create personal ‘sentencing codes’ to bring consistency and structure to their decision-making. These individualised frameworks aim to help them navigate the complexities of sentencing in their own cases; however, they cannot truly address the issue of systemic disparity.

Despite the development of personal guidelines, many professionals rely heavily on input from colleagues. This collegial support can provide valuable insights and feedback, helping to refine sentencing practices.

*Judge 2: "So, you go to your colleagues and ask, ‘Listen, I have a case here […] how much do you usually give for these types of cases? Where did it happen? Who was involved? And how much was it*—*like, if it was three kilos of marijuana or a kilo of cocaine?’ […] Because there’s no [pause] if you think about it, quantifying how much someone loves their spouse or whatever*—*it’s very difficult to translate that into specific numbers. So, it’s a struggle in that context."*

*Judge 5: "Yes, in my opinion, that's exactly it: [a junior judge will go] looking at all these judicial decisions, likely examining the actual circumstances and how they were evaluated. You seek more guidance from colleagues. They sometimes come to ask me what I think, and then often end up doing something quite different. But actually, it probably helps to discuss things with someone."*

These quotes highlight the reliance on collegial support to navigate the emotional complexities of sentencing. Judge 2 emphasizes the difficulty in quantifying subjective factors, such as emotional elements in a case, which leads to seeking advice from colleagues. This reflects the challenge of balancing personal judgment with the need for consistency in sentencing. Judge 5, while acknowledging the value of discussions, notes that colleagues sometimes make different decisions, revealing the limits of this approach in ensuring uniformity and is also consistent with academic findings in this area (Schultze et al., 2017).

The judges themselves highlighted this problem:

*Judge 5: "That is, the judges in [my court] compare themselves with each other but not with those in [other court] or [other court], you know, so then [it’s not very helpful]."*

Judges also reflected on their reliance on the prosecutorial recommendation, confirming the idea of anchoring, despite criticising the prosecution’s inconsistency at the same time.

*Judge 4: “What I’ve noticed is that, like it or not, a specific prosecutor's recommendation also serves as an anchor in my sentencing.”*

This comment highlights the psychological phenomenon of anchoring, where a previously presented value—such as a prosecutor’s suggested sentence—becomes a reference point that influences subsequent decisions (Bystranowski et al., 2021; Glöckner and Englich, 2015; Kim et al., 2015). Despite the criticism of prosecutorial inconsistency, Judge 4 acknowledges that this recommendation still plays a crucial role in shaping their sentencing decision. This reliance on external suggestions illustrates how, even when professionals recognise the lack of consistency in the system, they continue to be influenced by these external benchmarks.

Regardless, when presented with potential systemic solutions to address sentencing disparities, legal professionals exhibited a range of responses, reflecting their varying perspectives on the necessity and feasibility of changes.

Some professionals have actively sought to address sentencing inconsistencies on their own. For instance, one judge described creating a personal database of past cases to improve their own sentencing practices. At a broader level, the prosecution service, which had previously made only preliminary efforts to enhance consistency, has dedicated resources to a project aimed at improving sentencing practices *(Focus groups 1–3)*.

Their earlier initiatives, such as developing individual databases for each district prosecutorial office, were seen as somewhat beneficial but largely insufficient by most prosecutors *(Focus Groups 1–3)*. The new approach, which focuses on better collection and presentation of sentencing data and the development of general guidelines to promote consistency, received a mixed response. While it was generally accepted, it also generated some uncertainty. There was a clear rejection of mandatory structures, with preferences divided between sentencing tables and more flexible sentencing guidelines.

Judges, while agreeing that ‘something’ needs to be done, strongly opposed the idea of mandatory sentencing guidelines or rigid structures. They expressed concerns that such measures would unduly restrict their discretion and undermine the flexibility required to tailor sentences to individual cases. This apprehension reflects a belief that rigid standards could compromise their ability to deliver fair and individualised justice.

Moreover, many professionals were hesitant to embrace any kind of changes to the current system. They argued that there are no clear-cut solutions to the issue of sentencing disparity, aside from potentially increasing education on the topic over time. This reluctance reflects a broader scepticism about the effectiveness of systemic reforms and a preference for maintaining the status quo.

*Judge 4: “I probably would not use [a sentencing tool], but it’s something… something that I have not yet addressed, which is that I generally do not review case law. I do not look at case law, so I’d probably look at other things even less. But, for instance, if I think about it, it might be useful to review such materials to see what others are doing, what they think, and why they think that way, especially if there are good explanations provided. Just a database with a section and the corresponding sentences would not be very helpful to*” *e.”*

*Judge “: "I don’t know, I… [sigh] It’s hard to give a straightforward answer. My colleague and I were talking recently, and he mentioned that when passing a prison sentence in a high-profile case, he reviewed some… or all… past [similar] cases trying to calculate and compare things, and that seemed a bit odd to me. I’m not sure if that’s the right approach. You can’t always predict or categorise these things mathematically, you know, so I’m not sure…"*

Another important issue that emerged in our discussions, was the question of publicising sentencing data. Professionals agreed that such data should be accessible to prosecutors and judges, potentially through a unified platform for both groups. This would support more informed and consistent decision-making within the legal community. However, there was significant hesitation about sharing this data with the wider public. Concerns about transparency and privacy, as well as fears that public access to sentencing data could lead to misinterpretation and undermining of judicial discretion, contributed to this reluctance *(Focus groups 1–3)*.

In Slovenia, first-instance judgments are not currently publicised; however, an ongoing project aimed at automated anonymisation is expected to bring this about soon. Some professionals have noted that once this data becomes accessible, it is likely to be revealed eventually, whether through public channels or by private companies that might seize the opportunity to collect and analyse such information independently. Indeed, some solicitors and firms are already gathering their own sentencing data *(Focus group 1)*.

Amid these developments, there is a principled argument supporting the notion that sentencing data should be public knowledge, provided that data protection concerns are adequately addressed. A number of professionals strongly advocated for the release of such data, believing that transparency is crucial for ensuring accountability and fostering public trust in the judicial system.

Ultimately, the consequences of these issues and the uncertainty about how to address them have led to a growing sense of resignation among professionals. Faced with the limitations of their personal codes and the disillusionment with systemic support, many judges and prosecutors feel disheartened and powerless to effect meaningful change.

*Judge 2: "And so, I have to say, […] you become a bit desensitized over time… I mean, the first time I had to [sentence someone], I had a lump in my throat; now I don’t anymore. I also see more clearly when it makes sense to say something. Another thing is that you become desensitised in that sense as well. I’m talking about procedural aspects*—*what does it mean if a higher court corrects a criminal sentence? I don’t worry about that at all."*

## 5 Discussion

In this section, I want to bring together the theoretical background in which we started this paper and the empirical findings from the previous section. When we look at the thematic analysis, some thematic clusters emerge that can be useful in doing that. In the first one, we explore the emotional impact of recognising personal fallibility among legal professionals. This part draws on theories of cognitive dissonance and emotional responses to failure to explain how confronting evidence of one’s own inconsistencies leads to significant emotional distress. It opens the door to understanding the personal struggles that arise when professionals face direct evidence of their own mistakes.

The second part shifts the discussion to whether frustration stems more from the sentencing process’s inherent complexity or the disparity itself. By examining the challenges of standardising sentencing and the role of deliberate ignorance, this section highlights how professionals attempt to navigate the multifaceted nature of sentencing while grappling with systemic inconsistencies. This exploration reveals the broader frustrations and coping mechanisms that shape their approach to sentencing.

Finally, I address the professionals’ reluctance to embrace systemic reforms. This part delves into their concerns about potential changes, such as mandatory guidelines, and how these might impact their discretion and the delivery of justice. This part of the discussion links theoretical concepts about discretion and consistency with empirical observations of professionals’ resistance to structural changes.

### 5.1 Is it me?—facing personal fallibility

The emotional impact of acknowledging disparities in sentencing becomes particularly acute when legal professionals confront personal fallibility. Although the existence of sentencing disparities is widely acknowledged, it often does not evoke strong emotional reactions. However, the situation becomes markedly different when professionals face direct evidence of their own mistakes or inconsistencies. This realisation of personal imperfection is often accompanied by significant emotional distress, aligning with theories of cognitive dissonance (Festinger, 1957) and emotional responses to failure (Scheff, 1994).

In our study, judges and prosecutors exhibited a range of emotional reactions when confronted with their own inconsistencies. For instance, during workshops where they were shown the variation in their sentencing decisions, many displayed shock and disappointment through animated discussions, surprised facial expressions, and exclamations of disbelief. This personal involvement in the decision-making process heightened their emotional response compared to more general knowledge of disparity. Such reactions can be linked to the concept of cognitive dissonance, where professionals struggle to reconcile their self-image as fair and objective with the reality of their inconsistent decisions (Festinger, 1957).

Theoretical models of coping with failure highlight how individuals deal with personal imperfection. The responses observed in our study somewhat mirror these theoretical models (Dattner and Hogan, 2011). Some displayed extrapunitive reactions, where they attributed disparities to external factors or systemic issues rather than their own decisions. This aligns with the concept of deliberate ignorance, where professionals may downplay the significance of evidence showing discrepancies to avoid confronting their own biases or failures (Hertwig and Engel, 2021). Others exhibited what may be categorised as impunitive responses, which involve denial or rationalisations of the problem. For example, many emphasised the idea of individualisation as a reason for disparity, reflecting a reluctance to acknowledge the full extent of bias or error in their sentencing practices. This form of coping helps them maintain a sense of impartiality and fairness, even when faced with evidence that suggests otherwise.

Interestingly, intrapunitive responses, where professionals engage in excessive self-blame and criticism, were much less prevalent. Instead of internalizing blame, many judges and prosecutors were more inclined to criticize the system or other external factors rather than their own decision-making processes. Despite recognising the broader systemic issues, they often felt that their personal codes and methods were appropriate and justified. This tendency to externalise fault and rely on personal guidelines can be seen as a coping mechanism when the system does not provide adequate support or structure. However, this approach can be problematic as it does not strive towards achieving consistency across the board. By focusing on individual practices and dismissing systemic reforms, legal professionals may inadvertently perpetuate existing disparities rather than address the root causes of inconsistency (Kurlychek and Kramer, 2019).

Ultimately, while internalising blame was less common, and criticising the system was more acceptable, this divergence from systemic accountability highlights a broader issue. It underscores the tension between maintaining personal belief systems and the need for systemic consistency and fairness.

A related issue may also be the question of publicising sentencing data. While professionals agreed that such data should be made available to prosecutors and judges—potentially through a shared platform—they were much more hesitant about sharing it with the wider public. This reluctance was at least partly driven by concerns that public disclosure could expose their fallibility and undermine the integrity of the legal system. The fear of public scrutiny and the potential misuse of sentencing data may stem from a broader apprehension about being held accountable for their imperfections and inconsistencies in a system that is admittedly rather hostile against them (Pina-Sánchez and Plesničar, 2022). Some professionals expressed concerns that making such data public could lead to increased pressure and criticism, potentially impacting their professional standing and confidence (cf. Sunstein, 2007).

The push towards greater transparency is ongoing, with first-instance judgments in Slovenia expected to become public soon due to an automated anonymisation project. Some professionals acknowledged that, regardless of the judiciary’s stance, private entities might eventually publish this data. They argued that, considering these circumstances and the principle of transparency, any sentencing data should ideally be public knowledge, provided data protection considerations are adequately addressed.

### 5.2 Potato or potato?—sentencing complexity vs. disparity

While the evidence points to frustration with sentencing disparity, it is important to discern whether the frustration stems primarily from the disparity itself or from the broader challenges of sentencing. The complexity of sentencing encompasses numerous dimensions—legal, ethical, and personal—which makes it inherently difficult and often frustrating.

Professionals in our study expressed significant dissatisfaction with both the concept of disparity and the practice of sentencing. Many implied that while disparities are troubling, the broader frustration lies in the complexity and subjectivity of the sentencing process. Sentencing involves navigating a multitude of factors, including legal guidelines, personal judgments, and societal expectations, which can be overwhelming and difficult to standardise.

Efforts to mitigate this frustration included seeking advice from colleagues, developing personal sentencing codes, and striving to improve consistency through informal means. Despite these efforts, many professionals found that personal guidelines and collegial input were insufficient for achieving a systemic approach to consistency.

This aligns somewhat with the concept of deliberate ignorance, where professionals may acknowledge systemic flaws but choose to ignore them in favour of maintaining their current practices and beliefs (Hertwig and Engel, 2021).

The practice of deliberate ignorance allows professionals to cope with the emotional burden of recognising imperfections. By focusing on their individual practices and avoiding full engagement with systemic issues, they maintain a sense of fairness and impartiality. However, as in the previous section, this approach does not address the root causes of disparity and limits the effectiveness of systemic reforms.

### 5.3 Now what?—resistance to change

When presented with potential changes to address sentencing disparities, legal professionals demonstrated a clear reluctance. The primary concern was that mandatory guidelines or rigid structures would constrain their discretion and negatively impact their ability to deliver justice. This resistance reflects a broader apprehension about the implications of systemic reforms on their professional autonomy and the quality of justice.

There was a clear distinction among professionals regarding their attitudes towards potential changes and tools for addressing sentencing disparities. While all expressed a general wariness and scepticism towards systemic reforms, there were notable differences in how they approached the idea of guidance and tools. Some prosecutors and judges showed openness to the possibility of structured guidelines and expressed a willingness to welcome such changes, believing that they could improve consistency and fairness in sentencing. However, they also recognised the complexity of implementing these changes and the difficulties involved in tackling such systemic issues, which often led to discouragement and a reluctance to pursue further action.

At the level of prosecution, there were proactive efforts to develop tools and improve practices. Some senior prosecutors actively worked towards creating better data collection methods and general guidance to address inconsistencies. Despite these efforts, they found the process to be highly complex and challenging, which discouraged them from continuing their initiatives. The intricate nature of the task, coupled with the fear of diminishing their discretion, often led to a preference for incremental improvements rather than embracing comprehensive systemic reforms.

This reluctance to embrace mandatory sentencing guidelines or rigid frameworks stems from a fear that such measures would undermine the flexibility needed to tailor sentences to the unique circumstances of each case. Professionals expressed concerns that rigid standards could lead to unjust outcomes and stifle their ability to account for the nuances of individual cases. This apprehension is consistent with the theoretical understanding of the balance between discretion and consistency in sentencing (Bierschbach and Bibas, 2016; Pina-Sánchez, 2015; Plesničar, 2013).

Despite acknowledging the need for improvements, many professionals preferred to enhance their own practices within the existing system rather than adopt more structured approaches. They supported initiatives aimed at better data collection and general guidance but remained wary of mandatory structures that could restrict their discretion. This preference for maintaining the status quo reflects a deeper scepticism about the effectiveness of systemic reforms and a recognition of the inherent difficulties in implementing meaningful changes within the current framework.

Moreover, countering expectations in line with the action theory model of cognitive dissonance—according to which we would expect a strong inclination to resolve the dissonance (Harmon-Jones and Harmon-Jones, 2023), some sort of almost catatonic despondence was observed in some cases with a view that sentencing is inherently flawed. The potential pitfall of accepting that disparity in sentencing exists and not much can be done about it, is thus the risk of exacerbating disparities rather than mitigating them. Without a unified understanding of what constitutes appropriate sentencing, individual decision-makers’ attitudes and beliefs become more influential in shaping outcomes than the system ever intended (Hogarth, 1971). This variability in personal perspectives can lead to greater inconsistency and unpredictability in sentencing, further entrenching disparities within the system.

## 6 Conclusion

This paper has investigated the intricate dynamics between personal and systemic factors in addressing sentencing disparities, with a focus on the emotional and professional challenges encountered by judges and prosecutors. The difficulty of acknowledging one’s own failures is a universally recognised issue, but it becomes particularly pronounced in the legal profession, where decisions have profound and far-reaching consequences. The high stakes associated with sentencing underscore the importance and difficulty of recognising and addressing personal imperfections.

Existing research has acknowledged plenty of issues in making sentencing decisions, especially in avoiding disparity (Drápal, 2020; Lynch, 2019; Pina-Sánchez, 2015; Ulmer, 1997). A different strand of research has looked at the emotional toll of legal decision-making (e.g., Maroney, 2012) and specifically at the struggle to pacify the strong emotional charge with objectivity in legal decision-making (Bladini and Blix, 2022; Blix and Wettergren, 2019; Minissale, 2024). This study adds a new dimension by focusing specifically on the emotional struggles that arise when legal professionals are confronted with their own inconsistencies in sentencing.

Our findings reveal that while legal professionals nominally strive for objectivity in their sentencing practices, for example, looking for a more uniform approach aided by systematic data, they frequently grapple with the reality of their own fallibility. This struggle leads to significant emotional and professional stress, as evidenced by their varied reactions when confronted with personal inconsistencies. This tension between the ideal of objectivity and the subjective reality of decision-making aligns with theoretical frameworks on cognitive dissonance (Festinger, 1957) and emotional labour (Hochschild, 1983). Professionals—judges and prosecutors are no exception—experience substantial stress when faced with evidence of their own biases or errors (FitzGerald and Hurst, 2017; Sirriyeh et al., 2010), resulting in a range of responses from denial and defensiveness to self-criticism and anxiety.

The empirical findings align with these theoretical insights, demonstrating that the emotional responses to acknowledging imperfections are not merely abstract but have tangible effects on professionals’ well-being and their approach to sentencing. The study also highlights the limitations of personal guidelines and the role of deliberate ignorance in managing the emotional toll of decision-making. Legal professionals often develop personal “sentencing codes” and rely on collegial input to navigate the complexities of their roles. However, these individual efforts fall short of addressing systemic issues and can perpetuate disparities rather than mitigate them.

The reluctance of legal professionals to embrace systemic reforms, such as mandatory sentencing guidelines, reflects a deep-seated concern about preserving judicial discretion and flexibility in a system premised on the individualisation of punishment. This apprehension underscores the delicate balance between maintaining individualised justice and ensuring consistency in sentencing. The resistance to change suggests a preference for incremental improvements within the existing framework rather than overhauling the system in ways that could potentially constrain their discretion.

The findings emphasise the need for a nuanced understanding of sentencing that incorporates both emotional and systemic factors. Improved training on emotional and cognitive aspects of decision-making, along with enhanced support systems, seems crucial for helping legal professionals manage the pressures of their roles more effectively. Structured sentencing guidelines and clear support systems can contribute to a more consistent and fair legal process while also acknowledging the inherent human elements of decision-making, however finding the right balance is notoriously difficult.

Future research might continue to explore how legal professionals in different sentencing systems experience and manage their imperfections. Comparative studies of sentencing practices across various jurisdictions could provide valuable insights into how different frameworks impact professional behaviour and decision-making. Additionally, research on the effectiveness of potential reforms, such as enhanced training and support mechanisms, can help develop more effective strategies for addressing the emotional and systemic challenges faced by legal professionals.

Future research could further explore the long-term effects of these emotional challenges on legal professionals, as well as examine how similar issues play out in different legal systems. Additionally, there is a need for research into the effectiveness of potential reforms, such as structured sentencing guidelines and enhanced support systems, in mitigating these challenges. Understanding how legal professionals cope with these pressures in different cultural and legal contexts can provide valuable insights for the development of more effective policies and practices.

Addressing the emotional realities faced by legal professionals is crucial for maintaining the integrity and fairness of the justice system. By acknowledging and addressing these challenges, we can work towards a more humane and effective legal process. It is essential to balance the need for objectivity with the recognition that legal decision-making is inherently a human process, influenced by emotions and cognitive biases. Through thoughtful reforms and a commitment to supporting legal professionals, the justice system can continue to uphold the principles of fairness and justice upon which it is founded.

## Data Availability

The raw data supporting the conclusions of this article will be made available by the authors, without undue reservation.

## Footnotes

^1^All transcriptions were originally in Slovenian and have been translated into English by the author.
